# Correlation of *DJ-1*, *GDF15*, and *MFGE8* Gene Expression with Clinicopathological Findings in Gliomas and Meningiomas

**DOI:** 10.3390/ijms26189194

**Published:** 2025-09-20

**Authors:** Ayla Solmaz Avcikurt, Huseyin Utku Adilay, Omur Gunaldi, Sinem Gultekin Tosun, Salim Katar

**Affiliations:** 1 Department of Medical Biology, Faculty of Medicine, Balikesir University, Balikesir 10145, Turkey; 2 Department of Neurosurgery, Faculty of Medicine, Balikesir University, Balikesir 10145, Turkey; utkuadilay@hotmail.com (H.U.A.); salimkatar@gmail.com (S.K.); 3 Department of Neurosurgery, Basaksehir Cam and Sakura City Hospital, İstanbul 34480, Turkey; omurgunaldi@gmail.com

**Keywords:** *DJ-1*, mRNA expression, *GDF15*, glioma, meningioma, *MFGE8*

## Abstract

In light of the growing significance of molecular biomarkers in central nervous system tumours, in this study, we aimed to comprehensively and quantitatively analyze the mRNA expression levels of *DJ-1* (Parkinsonism-associated deglycase 7, *PARK7*), *GDF15* (Growth Differentiation Factor 15), and *MFGE8* (Milk Fat Globule-EGF Factor 8 Protein) in glioma and meningioma tissues and to thoroughly evaluate the associations between these gene expression profiles and clinicopathological parameters. Real-time PCR (qRT-PCR) analyses performed on tumour tissues obtained from a total of 27 glioma and 18 meningioma patients revealed that these three genes exhibited significantly elevated expression compared to control samples. Despite their different cellular origins, statistically significant positive correlations were observed between the expression levels of *DJ-1*, *GDF15*, and *MFGE8* and both tumour grade and the Ki-67 proliferation index (Ki-67 Pi) in both glioma and meningioma cases, indicating that higher gene expression is associated with increased tumour aggressiveness in both tumour types. Receiver operating characteristic (ROC) curve analyses further confirmed the diagnostic and prognostic potential of these genes. Additionally, protein–protein interaction networks involving the target genes were characterised, providing valuable insights into their molecular mechanisms. These findings suggest that *DJ-1*, *GDF15*, and *MFGE8* may play a role in the aggressiveness, invasion, and proliferation of gliomas and meningiomas. Moreover, integrating these genes as molecular biomarkers into tumour classification systems may provide a foundation for the development of personalised and targeted therapeutic strategies, although further studies are needed to support this.

## 1. Introduction

Central nervous system (CNS) tumours cover a highly heterogeneous group of neoplasms characterised by diverse morphological, molecular, and clinical features [1]. Among these, gliomas and meningiomas are particularly prominent because of their prevalence and the challenges they present in clinical management. Gliomas are the most common malignant CNS tumours, particularly affecting young and middle-aged adults. Arising from neuroepithelial progenitor cells (NPCs) in the embryonic neural tube and forebrain, they account for approximately 80% of all primary brain tumours [2,3]. In contrast, meningiomas, which are generally benign, represent the most frequent primary intracranial tumours in older adults. Originating from arachnoid cap cells of the arachnoid layer, their clinical course largely depends on tumour location and may lead to significant neurological deficits over time [4].

For many years, the classification of gliomas and meningiomas has been based on the histopathological criteria established by the World Health Organization (WHO). However, with the 2021 WHO classification, the approach based solely on morphological features has been extended to incorporate molecular biomarkers [5]. In this context, *IDH1* and *IDH2* mutations, 1p/19q codeletion, *TERT* promoter mutation, as well as *ATRX*, *TP53*, and *CDKN2A/B* alterations are of critical importance for gliomas [6,7]. In meningiomas, in addition to histopathological features, alterations in *NF2*, *AKT1*, *TRAF7*, *SMO*, *PIK3CA*, *KLF4*, and *SMARCE1* genes are recognised as key biomarkers in molecular classification [8,9]. This molecular approach largely overcomes the limitations of previous WHO classifications (interobserver variability, neglect of genetic heterogeneity, and exclusion of molecular markers), enabling more accurate predictions of tumour biological behaviour and clinical course. Therefore, a comprehensive understanding of the molecular heterogeneity of gliomas and meningiomas, along with the identification of novel diagnostic and prognostic biomarkers, is critical for both advancing tumour classification and improving clinical outcomes [10].

*DJ-1* (*PARK7*) plays a critical role in cellular defence mechanisms against oxidative stress and has been linked to poor prognosis across various cancer types [11]. Elevated *DJ-1* expression in gliomas has been reported to promote cell proliferation and enhance tumour aggressiveness through anti-apoptotic pathways [12,13]. Under normal conditions, *GDF15* (Growth Differentiation Factor 15) is expressed at low levels in most tissues; however, its expression significantly increases in pathological states such as tissue injury, inflammation, oxidative stress, and cancer, making it a potential biomarker in cancer patients [14]. It has been reported that *GDF15* is highly expressed in high-grade gliomas such as glioblastoma multiforme (GBM) and correlated with poor prognosis [15,16]. *MFGE8* (Milk Fat Globule-EGF Factor 8 Protein) contributes to extracellular matrix interactions and angiogenesis and has been implicated in tumour invasion and vascularization in both gliomas and meningiomas [17,18].

In this study, the expression levels of *DJ-1*, *GDF15*, and *MFGE8* genes were assessed in glioma and meningioma samples, and the resulting data were associated with clinical and pathological parameters, including sex, Ki-67 Pi, tumour diameter, and WHO tumour grade. The primary objective was to evaluate the potential of these genes as biomarkers and help provide a more precise biological classification of central nervous system tumours. Additionally, the findings are expected to provide a scientific foundation for the development of targeted therapeutic strategies.

## 2. Results

### 2.1. Demographic and Clinical Characteristics of Glioma and Meningioma Patients

A total of 27 glioma patients were included in the study. The mean age of the patients was 50.981 ± 13.303 years, with female patients having a mean age of 60.442 years and male patients 59.521 years. Among the cohort, 10 patients (37.037%) were female and 17 patients (62.963%) were male. Regarding tumour characteristics, 14 patients (51.852%) had tumours with a diameter of ≤6 cm, while 13 patients (48.148%) had tumours larger than 6 cm. The Ki-67 Pi was ≤22% in 12 patients (44.444%) and >22% in 15 patients (55.556%). Histopathological grading revealed that 8 patients (29.630%) were grade II, 3 patients (11.111%) were grade III, and 16 patients (59.259%) were grade IV. The pathological subtypes included astrocytoma in 6 patients (22.222%), oligodendroglioma in 5 patients (18.519%), and glioblastoma in 16 patients (59.259%). Tumour localization was predominantly frontal (9 patients, 33.333%), followed by parietal (8 patients, 29.630%), temporal (7 patients, 25.926%), and occipital (3 patients, 11.111%) regions. *IDH1* mutation analysis showed that 12 patients (44.444%) were positive for the mutation, whereas 15 patients (55.556%) were negative (Table 1).

A total of 18 meningioma patients were included in the study. The mean age of the patients was 59.873 ± 13.144 years, with female patients having a mean age of 59.413 years and male patients 60.332 years. Among the cohort, 12 patients (66.667%) were female and 6 patients (33.333%) were male. Regarding tumour characteristics, 8 patients (44.444%) had tumours with a diameter of ≤3 cm, while 10 patients (55.556%) had tumours larger than 3 cm. The Ki-67 Pi was ≤5% in 9 patients (50.000%) and >5% in 9 patients (50.000%). Histopathological grading revealed that 13 patients (72.222%) were grade I and 5 patients (27.778%) were grade II. The pathological subtypes included transitional meningioma in 5 patients (27.778%), angiomatous in 1 patient (5.556%), fibroblastic in 4 patients (22.222%), meningothelial in 2 patients (11.111%), psammomatous in 1 patient (5.556%), and atypical in 5 patients (27.778%). Secretory, metaplastic, and clear cell subtypes were not observed in this cohort. Tumour localization was predominantly frontal (8 patients, 44.444%), followed by parietal (5 patients, 27.778%), temporal (3 patients, 16.667%), and occipital (2 patients, 11.111%) regions (Table 2).

### 2.2. ROC Curve and AUC-Based Diagnostic Performance Analysis of DJ-1, GDF15, and MFGE8 Gene Expression in Glioma and Meningioma Tissues

Using RT-qPCR analysis, the expression levels of *DJ-1*, *GDF15*, and *MFGE8* genes were evaluated according to sex, tumour grade, tumour diameter, and Ki-67 Pi. mRNA expression levels were normalised relative to the control group, and results with *p* < 0.05 were considered statistically significant. In the glioma group, GBM (grade IV), which represents a prognostically critical subtype, was compared with lower-grade gliomas (grade II and III) in order to identify expression differences specific to high-grade malignancy. In meningiomas, grade I (benign) and grade II (atypical) tumours were compared to capture potential differences in clinical and biological behaviour. This approach aimed to evaluate not only the presence of these genes in tumours, but also their expression profiles in relation to tumour grade, thereby providing insights into their potential roles in tumour progression. Furthermore, the diagnostic discriminatory potential of these genes was assessed using ROC curve analyses. Discriminatory power was quantified by calculating the area under the curve (AUC), with genes exhibiting AUC values ≥ 0.7 considered to have diagnostic significance.

**Figure 1:**
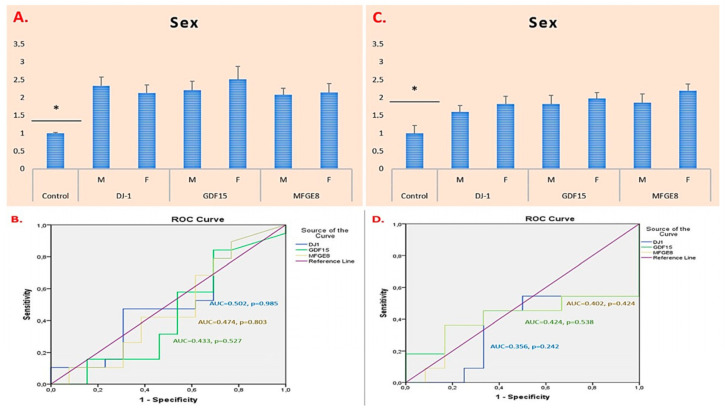
Sex-based mRNA expression of *DJ-1*, *GDF15*, and *MFGE8* in glioma and meningioma patients. (**A**) mRNA expression in glioma patients. (**B**) Glioma ROC/AUC. (**C**) mRNA expression in meningioma patients. (**D**) Meningioma ROC/AUC. M: Male, F: Female. * *p* < 0.05 was considered statistically significant.

The expression levels of the *DJ-1*, *GDF15*, and *MFGE8* genes in glioma patients were elevated compared to those in the control group. Despite the high expression levels of these genes in glioma cases, no statistically significant differences were observed between male and female patients (*DJ-1*, *GDF15*, and *MFGE8*: *p* = 0.563, *p* = 0.490, and *p* = 0.831, respectively). Furthermore, ROC analyses performed for glioma cases revealed that the AUC values for the *DJ-1*, *GDF15*, and *MFGE8* genes were all below 0.7, with *p*-values above 0.05, indicating that these genes do not have sufficient diagnostic discriminatory power based on patient sex in glioma (Figure 1A,B).

The expression levels of the *DJ-1*, *GDF15*, and *MFGE8* genes in meningioma patients were elevated compared to those in the control group. No statistically significant differences were observed between male and female patients in terms of *DJ-1*, *GDF15*, and *MFGE8* gene expression levels (*DJ-1*, *GDF15*, and *MFGE8*: *p* = 0.438, *p* = 0.603, and *p* = 0.281, respectively). ROC analyses for meningioma cases revealed that the AUC values for these genes were all below 0.7, with *p*-values above 0.05, indicating that *DJ-1*, *GDF15*, and *MFGE8* do not have sufficient diagnostic discriminatory power based on patient sex in meningioma (Figure 1C,D).

**Figure 2:**
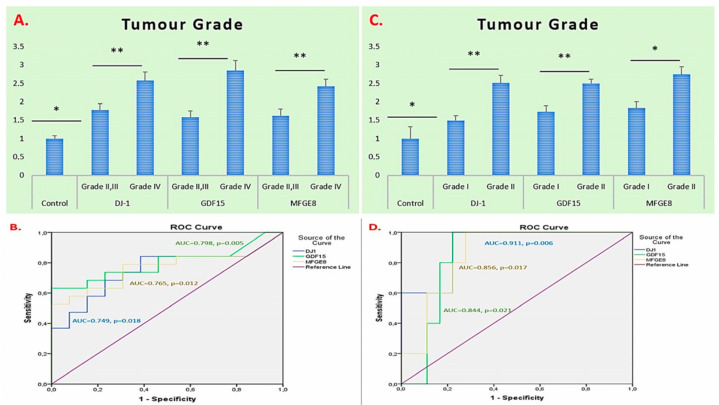
Tumour-grade-based mRNA expression of *DJ-1*, *GDF15*, and *MFGE8* in glioma and meningioma patients. (**A**) mRNA expression in glioma patients. (**B**) Glioma ROC/AUC. (**C**) mRNA expression in meningioma patients. (**D**) Meningioma ROC/AUC. * *p* < 0.05 and ** *p* < 0.01 was considered statistically significant.

Evaluation of tumour grades in glioma patients revealed that the expression levels of the *DJ-1*, *GDF15*, and *MFGE8* genes were increased compared to those in the control group. The most prominent increase was observed in the *GDF15* gene, and the expression levels of *DJ-1* and *MFGE8* were also significantly higher in high-grade tumours (Grade IV) compared to low- to intermediate-grade tumours (Grade II–III) (*p* = 0.016, *p* = 0.001, and *p* = 0.006 for *DJ-1*, *GDF15*, and *MFGE8*, respectively). ROC analyses were performed to evaluate the diagnostic discriminatory potential of *DJ-1*, *GDF15*, and *MFGE8* gene expressions based on tumour grade. The AUC values for all three genes were above 0.7, and the *p*-values were less than 0.05, indicating statistical significance (Figure 2A,B).

In meningioma patients, the expression levels of the *DJ-1*, *GDF15*, and *MFGE8* genes were significantly higher in Grade II tumours compared to Grade I tumours. The greatest increase was observed in the MFGE8 gene (*p* = 0.001, *p* = 0.001, and *p* = 0.012 for *DJ-1*, *GDF15*, and *MFGE8*, respectively). ROC analyses were also performed to assess the diagnostic discriminatory potential of these genes based on tumour grade, showing AUC values above 0.7 with *p*-values less than 0.05, indicating statistical significance (Figure 2C,D).

**Figure 3:**
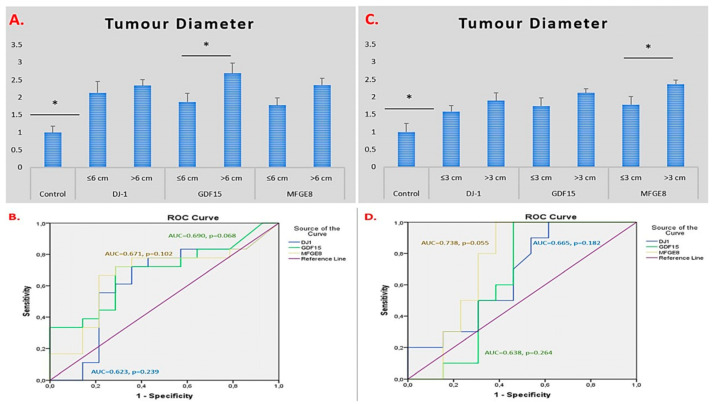
Tumour-diameter-based mRNA expression of *DJ-1*, *GDF15*, and *MFGE8* in glioma and meningioma patients. (**A**) mRNA expression in glioma patients. (**B**) Glioma ROC/AUC. (**C**) mRNA expression in meningioma patients. (**D**) Meningioma ROC/AUC. * *p* < 0.05 was considered statistically significant.

When tumour diameter was evaluated in glioma patients, the expression levels of the *DJ-1*, *GDF15*, and *MFGE8* genes were found to be elevated compared to those in the control group. Gene expression levels of *DJ-1*, *GDF15*, and *MFGE8* were increased in tumours larger than 6 cm in diameter, with the most prominent and statistically significant increase observed in the *GDF15* gene (*p* = 0.556, *p* = 0.042, and *p* = 0.053 for *DJ-1*, *GDF15*, and *MFGE8*, respectively). ROC analyses were performed to evaluate the diagnostic performance of the *DJ-1*, *GDF15*, and *MFGE8* genes in discriminating tumour diameter. In glioma cases, the AUC values for all three genes were below 0.7, and their *p*-values were above 0.05, indicating that they do not have sufficient discriminatory power based on tumour size (Figure 3A,B).

In meningioma patients, the expression levels of *DJ-1*, *GDF15*, and *MFGE8* genes were elevated in tumours larger than 3 cm compared to smaller tumours and the control group. The most prominent and statistically significant increase was observed in the *MFGE8* gene (*p* = 0.277, *p* = 0.167, and *p* = 0.044 for *DJ-1*, *GDF15*, and *MFGE8*, respectively). ROC analyses showed that the AUC values for *DJ-1* and *GDF15* were below 0.7, with *p*-values above 0.05, whereas the *MFGE8* gene demonstrated an AUC value above 0.7 with a *p*-value below 0.05, indicating that only *MFGE8* has potential diagnostic discriminatory power for tumour diameter in meningioma (Figure 3C,D).

**Figure 4:**
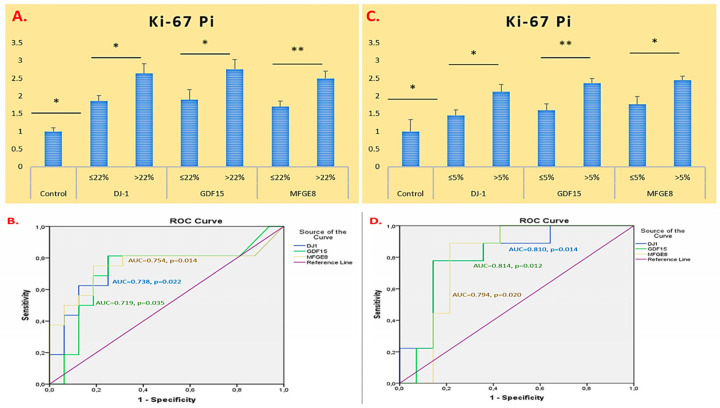
Ki-67-index-based mRNA expression of *DJ-1*, *GDF15*, and *MFGE8* in glioma and meningioma patients. (**A**) mRNA expression in glioma patients. (**B**) Glioma ROC/AUC. (**C**) mRNA expression in meningioma patients. (**D**) Meningioma ROC/AUC. * *p* < 0.05 and ** *p* < 0.01 was considered statistically significant.

When the Ki-67 Pi was evaluated in glioma patients, the expression levels of the *DJ-1*, *GDF15*, and *MFGE8* genes were elevated compared to those in the control group. All three genes were significantly higher in cases with a Ki-67 Pi value > 22% compared to those with Ki-67 Pi ≤ 22% (*DJ-1*, *GDF15*, and *MFGE8*; *p* = 0.015, *p* = 0.035, and *p* = 0.004, respectively). Among these genes, the most pronounced increase was observed in the *GDF15* gene. ROC analyses related to Ki-67 Pi showed that all three genes had AUC values above 0.7 and *p*-values below 0.05, indicating statistically significant discriminatory potential based on proliferation index (Figure 4A,B).

In meningioma patients, *DJ-1*, *GDF15*, and *MFGE8* gene expression levels were significantly elevated in tumours with Ki-67 Pi > 5% compared to those with Ki-67 Pi ≤ 5% (*DJ-1*, *GDF15*, and *MFGE8*; *p* = 0.015, *p* = 0.003, and *p* = 0.012, respectively). Among these increases, the highest expression level was observed in the *MFGE8* gene. ROC analyses related to Ki-67 Pi demonstrated that all three genes had AUC values above 0.7 and *p*-values below 0.05, indicating statistically significant discriminatory potential based on proliferation index in meningioma (Figure 4C,D).

**Figure 5:**
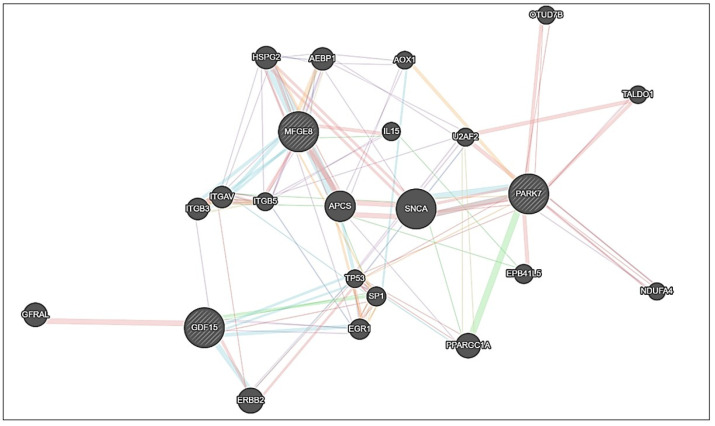
A protein–protein interaction network of *DJ-1*, *GDF15*, and *MFGE8* constructed using GeneMANIA. The lines connecting the proteins represent different types of interactions, such as physical interactions, co-expression, co-localization, shared protein domains, and pathway associations, as predicted by GeneMANIA.

In this study, the gene expression profiles of *DJ-1*, *GDF15*, and *MFGE8* in tissue samples obtained from glioma and meningioma patient groups were found to be elevated compared to those in the control group. To comprehensively elucidate the biological interactions and potential functional roles of these genes in tumour pathophysiology, a protein–protein interaction (PPI) network was generated using the GeneMANIA platform (https://genemania.org/), with these genes serving as seed nodes. Analysis of the constructed network suggests that *DJ-1*, *GDF15*, and *MFGE8* may occupy central hub positions and potentially exhibit functional associations with a wide range of interacting genes and proteins (Figure 5).

## 3. Discussion

In this study, the mRNA expression profiles of *DJ-1*, *GDF15*, and *MFGE8* genes were analyzed in glioma and meningioma tissues, and their potential associations with clinicopathological parameters were evaluated. Within the glioma cohort, the prognostically critical subtype GBM (grade IV) was compared with lower-grade gliomas (grade II–III) to explore molecular heterogeneity reflecting differences in biological behaviour. While grade II–III gliomas generally exhibit a slower clinical course and more favourable prognosis, grade IV glioblastomas may display more aggressive biological behaviour, higher proliferative capacity, invasive growth, and treatment resistance. Comparison of gene expression levels between these groups may provide insights into the potential roles of *DJ-1*, *GDF15*, and *MFGE8* in tumour progression, malignant transformation, and poor prognosis. Similarly, in the meningioma cohort, grade I (benign) and grade II (atypical) tumours were compared. Grade I meningiomas typically show slower growth and favourable prognosis, whereas grade II meningiomas may exhibit increased recurrence risk and more aggressive clinical behaviour. Therefore, examining gene expression levels across these subgroups may offer a framework for assessing the potential of *DJ-1*, *GDF15*, and *MFGE8* to discriminate between benign and atypical meningiomas and their possible functional roles in tumour biology.

In glioma tissues, the expression levels of *DJ-1*, *GDF15*, and *MFGE8* did not show a significant correlation with patient sex. When evaluated in terms of tumour size, only *GDF15* expression demonstrated a statistically significant association, whereas no such relationship was observed for *DJ-1* and *MFGE8*.

In meningioma tissues, the expression levels of *DJ-1*, *GDF15*, and *MFGE8* were not significantly associated with patient sex. Regarding tumour size, only *MFGE8* expression showed a significant correlation, while *DJ-1* and *GDF15* did not exhibit statistically meaningful associations.

These findings suggest that the expression of these genes may be primarily related to biological features such as tumour progression, proliferative capacity, and molecular subtypes. Future studies with larger patient cohorts and inclusion of different tumour subtypes could allow a more detailed evaluation of potential relationships between gene expression and clinical parameters. Additionally, investigating the proteomic effects of *DJ-1*, *GDF15*, and *MFGE8*, as well as their interactions with the tumour microenvironment, could provide a more comprehensive understanding of the clinical relevance of their expression profiles.

In gliomas, statistically significant correlations were identified between the expression levels of *DJ-1*, *GDF15*, and *MFGE8* and both tumour grade and the Ki-67 Pi. *DJ-1* expression is associated with brain tumours. In patients with astrocytoma, *DJ-1* was highly expressed in 92.8% of cases, and this has been suggested to be associated with disease aggressiveness and reduced overall survival in these patients [19]. Furthermore, *DJ-1* expression has been reported to be upregulated in 85% of glioblastoma patients and 48.5% of medulloblastoma patients [20,21]. *DJ-1* expression has also been found to correlate with increased expression of p-protein kinase B (AKT) and Ki-67, potentially affecting patient survival. Similarly, previous studies have shown that *GDF15* may promote proliferation in glioblastoma cells [22], support tumour growth by stimulating angiogenesis, and be associated with adverse prognostic outcomes when highly expressed [23]. Regarding *MFGE8*, data from the Human Protein Atlas indicate that its expression may positively correlate with tumour grade in glioma tissues [24]. Experimental studies have also demonstrated that *MFGE8* may contribute to glioma progression by modulating the tumour microenvironment and promoting angiogenesis [18]. Consistent with these observations, our study identified statistically significant correlations between the expression levels of *DJ-1*, *GDF15*, and *MFGE8* and both tumour grade and the Ki-67 Pi in glioma tissues. These findings support the potential functional roles of these genes in glioma pathophysiology and their involvement in tumour progression and cellular proliferation mechanisms. Nevertheless, these results should be interpreted with caution, and further validation in advanced studies is required to clarify their molecular mechanisms and potential clinical significance.

In meningiomas, *GDF15* gene expression was significantly associated with tumour grade and the Ki-67 Pi. These findings may indicate the potential prognostic value of *GDF15* in these tumours. Additionally, the observed associations of *DJ-1* and *MFGE8* expression with histological grade and proliferative activity indicate that these genes may contribute to the evaluation of malignancy potential in meningiomas. However, the relatively limited correlation between *MFGE8* expression and tumour grade suggests that the former’s role may be complex and multifactorial. Supporting this notion, literature reports have described *MFGE8* as having diverse functions, including angiogenesis and immune modulation within the tumour microenvironment [25,26]. In this context, *MFGE8* may interact not only with tumour-intrinsic factors such as histologic grade but also with microenvironmental components, potentially influencing meningioma behaviour in a broader biological framework. Therefore, further comprehensive studies are required in order to elucidate the prognostic significance and mechanistic roles of *MFGE8* in meningiomas. Although the critical role of *GDF15* in tumour progression and the regulation of the immune microenvironment has been demonstrated in glioma subtypes [22], the limited evidence supporting its involvement in tumour growth and invasion in meningiomas increases the originality of our study and its potential contribution to the literature. Similarly, the observed association between *DJ-1* and histological grade and proliferative index in meningiomas aligns with its established roles in cell proliferation, oxidative stress response, and tumour progression, as reported in previous studies [25,27]. Moreover, *DJ-1* has been shown to facilitate cell migration and invasion, thereby contributing to the malignant potential of various tumour types [13]. Based on these findings, the hypothesis that *DJ-1* expression may serve as an indicator of malignancy in meningiomas is well supported by the current literature.

In our study, a protein–protein interaction (PPI) network analysis was conducted using the GeneMANIA database to further evaluate the potential roles of *DJ-1* (*PARK7*), *GDF15*, and *MFGE8* genes in glioma and meningioma pathogenesis. This analysis can provide insights into the possible functions of these genes in intracellular and extracellular signalling pathways and their associations with tumour biology. *DJ-1* was found to potentially interact with proteins primarily involved in oxidative stress response and mitochondrial function, including TP53, SNCA, U2AF2, TALDO1, and NDUFA4. Specifically, TP53, as a tumour suppressor gene, has been associated with tumour progression and prognosis in gliomas and meningiomas, with mutations and reduced expression linked to aggressive tumour behaviour and poor prognosis [28]. The interaction of *DJ-1* with SNCA is related to neurodegenerative processes, and increased SNCA expression has been reported in certain glioma subgroups [29]. Its association with U2AF2 may influence RNA processing and gene expression; however, data on U2AF2’s role in gliomas remain limited [30]. Interactions with TALDO1 and NDUFA4 suggest potential involvement in glycolysis, energy metabolism, and mitochondrial functions, which are frequently altered in gliomas [31,32].

The *GDF15* gene may interact with ERBB2, TP53, and SP1, which are involved in extracellular signal transmission and regulation of immune responses in the tumour microenvironment. Potential interactions with ERBB2 and TP53 may support roles in cell proliferation, apoptosis, and tumour progression, and ERBB2 expression has been reported to increase in gliomas [33,34]. Interaction with SP1 may exert additional regulatory effects on gene transcription and signalling; high SP1 expression in meningiomas has been associated with tumour grade and malignancy, suggesting a possible prognostic biomarker role [35].

Similarly, *MFGE8* may interact with integrin subunits ITGB3, ITGB5, and ITGAV, and also with IL15 and AEBP1 proteins, which are associated with extracellular matrix and immune response processes. Interactions with integrin subunits may influence cell adhesion and migration, while associations with IL15 and AEBP1 may affect immune responses and cell differentiation. ITGB3 and ITGB5 have been reported to modulate apoptosis and proliferation in glioma cells, contributing to tumour progression [36,37]. IL15 is considered a potential therapeutic modulator of immune response in gliomas [38]. Elevated AEBP1 expression has been linked to proliferation, migration, and invasion in glioblastomas [39]. ITGAV has been associated with poor prognosis and immune cell infiltration in gliomas, potentially affecting tumour immune responses and progression [40].

Overall, elevated expression levels of *DJ-1*, *GDF15*, and *MFGE8* in glioma and meningioma samples, together with the observed PPI network connections, provide a framework for exploring their potential roles in tumour pathogenesis. Integrating PPI-based findings with gene expression data may contribute to understanding the possible mechanistic roles of *DJ-1*, *GDF15*, and *MFGE8* in glioma and meningioma biology.

In light of these findings, it was observed that the expression levels of *DJ-1*, *GDF15*, and *MFGE8* genes may be associated with certain key clinical parameters in glioma and meningioma samples, which originate from different cellular lineages. In particular, the correlations observed with tumour grade and the Ki-67 Pi may indicate that these genes could play a potentially critical role as molecular markers in tumour biology.

Our study has certain limitations. Firstly, due to the relatively small sample size and the high heterogeneity of tumours, comprehensive molecular classifications regarding common genetic alterations (e.g., *IDH2*, *TP53*, 1p/19q codeletion, *TERT* promoter mutation) could not be performed in the glioma cohort. This limitation may reduce the generalizability of inferences drawn from gene expression and other molecular findings. In the meningioma cohort, genetic analyses were not conducted, and tumour classification was based solely on histopathological evaluation according to the WHO 2021 criteria. Therefore, molecular validation of meningioma findings (e.g., *NF2*, *AKT1*, *TRAF7*) and an increase in both sample size and tumour subtype diversity are important for enhancing the reliability and generalizability of future studies.

In our study, the Ki-67 Pi and tumour size were categorised for glioma and meningioma samples based on previously defined and widely used cut-off values reported in the literature. However, Ki-67 Pi values ranged from 2 to 6% and tumour diameters from ≤3 cm to >3 cm in meningiomas, whereas in gliomas, Ki-67 Pi values ranged from 2 to 80% and tumour diameters from ≤6 cm to >6 cm. This variability highlights the challenges in establishing a single common cut-off value for both tumour types. Considering literature-based cut-off values, it is plausible that the diagnostic potential of these analyses may be context-dependent. Therefore, evaluating the Ki-67 Pi and tumour size as continuous parameters in future studies and testing different cut-off values in larger patient cohorts may enhance the clinical and scientific validity of the findings.

According to TCGA and CGGA datasets, *DJ-1*, *GDF15*, and *MFGE8* were found to be highly expressed in gliomas. Due to the absence of survival data for meningiomas and the limited size of the patient cohort, survival analyses could not be performed in this study. However, in future studies with larger patient groups, the integration of publicly available datasets (TCGA, CGGA) may allow a clearer elucidation of the relationship between these genes and patient survival and contribute to validating the prognostic value of the findings.

Furthermore, future studies should aim to support these results through protein expression analyses and to investigate the functional implications of protein–protein interactions associated with the target genes within relevant signalling pathways. Expanding and deepening the scope of such studies could provide more robust and consistent evidence regarding the biomarker potential of *DJ-1*, *GDF15*, and *MFGE8*. Ultimately, a better understanding of the relationships between tumour biology and clinical parameters may contribute to improved diagnosis, prognostic assessment, and the development of personalised therapeutic strategies.

## 4. Materials and Methods

### 4.1. Ethical Statement Declaration

This study was conducted in collaboration with the Neurosurgery Clinic of the Health Sciences University, Bakırköy Prof. Dr. Mazhar Osman Mental Health and Neurological Diseases Training and Research Hospital, and the Medical Biology and Genetics Laboratory of the Balıkesir University Faculty of Medicine. All patients and their relatives provided written informed consent after receiving detailed information about the study.

Ethical approval for this study was obtained from the Clinical Research Ethics Committee of the Balıkesir University Faculty of Medicine. The following ethics committee approvals were granted: 2017/169 for glioma studies on *DJ-1* gene expression, 2017/170 for meningioma studies, 2018/07 for glioma studies on *GDF15* gene expression, 2018/08 for meningioma studies, 2018/05 for glioma studies on *MFGE8* gene expression, and 2017/168 for meningioma studies. All tissue samples were collected in compliance with the principles established by the relevant ethics committee, and all procedures were conducted in accordance with the Declaration of Helsinki.

### 4.2. Collection of Tumour Tissue Samples

This study included 27 patients diagnosed with astrocytoma, oligodendroglioma (WHO grade II–III), and glioblastoma (WHO grade IV), and 18 patients diagnosed with meningioma (WHO grade I–II), all of whom were treated at the Neurosurgery Clinic of Bakırköy Prof. Dr. Mazhar Osman Mental Health and Neurological Diseases Training and Research Hospital in 2017. Patients with severe systemic or metabolic diseases, hyperproliferative or infectious conditions, impaired renal function, additional malignancies, recent surgical history, or those who declined participation were excluded from the study. The control group consisted of five patients who underwent anterior temporal lobectomy for mesial temporal lobe epilepsy and met the exclusion criteria.

Sociodemographic data, including age, sex, and comorbidities, were recorded for glioma and meningioma patients; tumour classification, size, and WHO grade were determined at a single-centre pathology unit using standard histological methods. The Ki-67 Pi was assessed by immunohistochemical staining and calculated as the percentage of positively stained cells relative to the total number of cells. The maximum dimensions of the lesions were measured on pathological specimens in the sagittal, coronal, or axial planes. All diagnoses were confirmed according to the WHO 2021 criteria. Additionally, the isocitrate dehydrogenase (IDH1) mutation status was evaluated in glial tumours. Tumour and control tissue samples obtained via surgical resection were stored at −80 °C for subsequent gene expression analyses.

### 4.3. RNA Isolation and qRT-PCR

Gene expression levels of *DJ-1*, *GDF15*, and *MFGE8* were evaluated via qRT-PCR in the glioma (n = 27), meningioma (n = 18), and control (n = 5) tissue samples that had been stored at −80 °C in the Medical Biology and Genetics Laboratory of the Faculty of Medicine at Balıkesir University. Gene expression was analyzed in relation to pathological characteristics, including tumour diameter, tumour grade, and Ki-67 Pi, as well as sociodemographic variables such as age and sex.

Tumour diameter was categorised as ≤6 cm and >6 cm for glioma patients [41] and ≤3 cm and >3 cm for meningioma patients [42]. Tumour grading was classified as Grade II–III and Grade IV for gliomas [43] and as Grade I and Grade II for meningiomas [44]. The Ki-67 Pi was stratified as ≤22% and >22% for glioma cases [45] and ≤5% and >5% for meningioma cases [46].

Total RNA was isolated using the QIAzol Lysis Reagent (Qiagen GmbH, Hilden, Germany), following the manufacturer’s protocol. RNA concentration (ng/µL) and purity (OD260/280) were measured using a NanoDrop device (Thermo Scientific, Wilmington, DE, USA). A total of 1 µg of RNA was used for complementary DNA (cDNA) synthesis. cDNA synthesis was performed using the Transcriptor High Fidelity cDNA Synthesis Kit (Roche Diagnostics GmbH, Mannheim, Germany) in accordance with the manufacturer’s instructions. For each reaction, 1 µg of total RNA was mixed with 2 µL of random hexamer primers and PCR-grade water, reaching a final volume of 11.4 µL. The mixture was incubated at 65 °C for 10 min. Subsequently, the following components were added to the mixture: 4 µL of 5× Reverse Transcriptase Reaction Buffer, 0.5 µL of Protector RNase Inhibitor (40 U/µL), 2 µL of 10 mM dNTP mix, 1 µL of DTT, and 1.1 µL of Transcriptor High-Fidelity Reverse Transcriptase, resulting in an additional volume of 8.6 µL. After brief centrifugation, the total volume was adjusted to 20 µL. cDNA synthesis was carried out in a thermal cycler under the following conditions: incubation at 29 °C for 10 min, 48 °C for 60 min, and 85 °C for 5 min for enzyme inactivation.

qRT-PCR analysis was performed using the Applied Biosystems 7500 Fast Real-Time PCR System and TaqMan probes (Applied Biosystems, Foster City, CA, USA). Specific TaqMan Gene Expression Assays were employed for the reference gene *β-actin* (ACTB, Hs99999903_m1) and the target genes: *DJ-1* (Hs00994893_g1), *GDF15* (Hs00171132_m1), and *MFGE8* (Hs00983890_m1). Each reaction was conducted in a total volume of 20 µL, containing 1–100 ng of cDNA, 20× TaqMan Gene Expression Assay, 2× TaqMan Gene Expression Master Mix, and RNase-free water. Reactions were run for 40 cycles under standard cycling conditions. Gene expression data were analysed using the Applied Biosystems 7500 Fast Real-Time PCR System, 7500 Software v2.3. The ACTB gene served as an internal control for normalization. Relative gene expression levels were calculated using the 2^−ΔΔCT^ method [47].

### 4.4. Statistical Analysis

Statistical analyses were performed using SPSS version 20 (IBM Corp., Armonk, NY, USA). The normality of the data distribution was assessed using the Shapiro–Wilk test, and the homogeneity of variance was evaluated using Levene’s test. Differences between the means of multiple groups were analyzed using one-way analysis of variance (ANOVA), and Tukey’s post hoc test was applied in cases where significant differences were found. Comparisons between two groups were performed using Student’s *t*-test. A *p*-value of <0.05 was considered statistically significant. ROC curve analyses were conducted, and Area Under the Curve (AUC) values were calculated to assess the diagnostic discriminative potential of *DJ-1*, *GDF15*, and *MFGE8* gene expression levels in relation to variables such as sex, tumour grade, tumour diameter, and Ki-67 Pi. Genes with AUC values of ≥0.7 were considered to have diagnostic significance.

## 5. Conclusions

In this study, significant and marked increases in the expression levels of the *DJ-1*, *GDF15*, and *MFGE8* genes were detected in both glioma and meningioma tissues. These findings suggest that these genes may hold potential as molecular biomarkers. However, for these genes to be effectively and reliably evaluated in clinical practice, the limitations noted in this study need to be addressed, their expression levels should be validated at the protein level, and their mechanistic roles in tumour development and progression should be investigated more comprehensively.

## Figures and Tables

**Table 1:** Clinical parameters and demographic distribution of glioma patients.

Parameter	Glioma (n = 27)
Mean Age (year)	50.981 ± 13.303
	Female: 60.442
	Male: 59.521
Sex, n (%)	Female: 10 (37.037)
	Male: 17 (62.963)
Tumour Diameter, n (%)	≤6 cm: 14 (51.852)
	>6 cm: 13 (48.148)
Ki-67 Pi, n (%)	≤22%: 12 (44.444)
	>22%: 15 (55.556)
Grade, n (%)	II: 8 (29.630)
	III: 3 (11.111)
	IV: 16 (59.259)
Pathological subtype, n (%)	Astrositoma: 6 (22.222)
	Oligodendroglioma: 5 (18.519)
	Glioblastoma: 16 (59.259)
Localization	Frontal: 9 (33.333)
	Occipital: 3 (11.111)
	Parietal: 8 (29.630)
	Temporal: 7 (25.926)
*IDH1* mutation status, (+/−)	Absent: 15 (55.556)
	Present: 12 (44.444)

**Table 2:** Clinical parameters and demographic distribution of meningioma patients.

Parameter	Meningioma (n = 18)
Mean Age (year)	59.873 ± 13.144
	Female: 59.413
	Male: 60.332
Sex, n (%)	Female: 12 (66.667)
	Male: 6 (33.333)
Tumour Diameter, n (%)	≤3 cm: 8 (44.444)
	>3 cm: 10 (55.556)
Ki-67 Pi, n (%)	≤5%: 9 (50.000)
	>5%: 9 (50.000)
Grade, n (%)	I: 13 (72.222)
	II: 5 (27.778)
Pathological subtype, n (%)	Transitional: 5 (27.778)
	Angiomatous: 1 (5.556)
	Secretory: 0 (00.00)
	Fibroblastic: 4 (22.222)
	Meningothelial: 2 (11.111)
	Metaplastic: 0 (00.00)
	Psammomatous: 1 (5.556)
	Clear cell: 0 (00.00)
	Atypical: 5 (27.778)
Localization	Frontal: 8 (44.444)
	Occipital: 2 (11.111)
	Parietal: 5 (27.778)
	Temporal: 3 (16.667)

## Data Availability

The data used to support the findings of this study are available from the corresponding author upon request.

## Abbreviations

The following abbreviations are used in this manuscript:

*ATRX*	Alpha-thalassemia X-linked intellectual disability
*DJ-1*	Parkinsonism associated deglycase 7/PARK7
*GDF15*	Growth Differentiation Factor 15
*IDH1/2*	Isocitrate Dehydrogenase 1/2
*KLF4*	Kruppel-Like Factor 4
*MFGE8*	Milk Fat Globule-EGF Factor 8 Protein
*NF2*	Neurofibromin 2
*SMO*	Smoothened Frizzled Class Receptor
*TERT*	Telomerase Reverse Transcriptase
*TP53*	Tumour Protein 53
*TRAF7*	TNF Receptor Associated Factor 7

## References

[1] Ramkissoon S. (2014). Surgical pathology of neoplasms of the central nervous system. Pathobiology of Human Disease.

[2] Schaff L.R., Mellinghoff I.K. (2023). Glioblastoma and other primary brain malignancies in adults: A review. JAMA.

[3] Zuchero J.B., Barres B.A. (2015). Glia in mammalian development and disease. Development.

[4] Huntoon K., Toland A.M.S., Dahiya S. (2020). Meningioma: A review of clinicopathological and molecular aspects. Front. Oncol..

[5] Louis D.N., Perry A., Wesseling P., Brat D.J., Cree I.A., Figarella-Branger D., Hawkins C., Ng H.K., Pfister S.M., Reifenberger G. (2021). The 2021 WHO classification of tumors of the central nervous system: A summary. Neuro Oncol..

[6] Louis D.N., Holland E.C., Cairncross J.G. (2001). Glioma classification: A molecular reappraisal. Am. J. Pathol..

[7] Ceccarelli M., Barthel F.P., Malta T.M., Sabedot T.S., Salama S.R., Murray B.A., Morozova O., Newton Y., Radenbaugh A., Pagnotta S.M. (2016). Molecular profiling reveals biologically discrete subsets and pathways of progression in diffuse glioma. Cell.

[8] Eckel-Passow J.E., Lachance D.H., Molinaro A.M., Walsh K.M., Decker P.A., Sicotte H., Pekmezci M., Rice T., Kosel M.L., Smirnov I.V. (2015). Glioma groups based on 1p/19q, IDH, and TERT promoter mutations in tumors. N. Engl. J. Med..

[9] Wang J.Z., Landry A.P., Raleigh D.R., Sahm F., Walsh K.M., Goldbrunner R., Yefet L.S., Tonn J.C., Gui C., Ostrom Q.T. (2024). Meningioma: International consortium on meningiomas consensus review on scientific advances and treatment paradigms for clinicians, researchers, and patients. Neuro Oncol..

[10] Louis D.N., Perry A., Reifenberger G., von Deimling A., Figarella-Branger D., Cavenee W.K., Ohgaki H., Wiestler O.D., Kleihues P., Ellison D.W. (2016). The 2016 World Health Organization classification of tumors of the central nervous system: A summary. Acta Neuropathol..

[11] Hsieh A.L., Bi W.L., Ramesh V., Brastianos P.K., Plotkin S.R. (2024). Evolving concepts in meningioma management in the era of genomics. Cancer.

[12] Wilson M.A. (2011). The role of cysteine oxidation in *DJ-1* function and dysfunction. Antioxid. Redox Signal..

[13] Olivo E., La Chimia M., Ceramella J., Catalano A., Chiaradonna F., Sinicropi M.S., Cuda G., Iacopetta D., Scumaci D. (2022). Moving beyond the tip of the iceberg: *DJ-1* implications in cancer metabolism. Cells.

[14] Wang C., Fang M., Zhang M., Li W., Guan H., Sun Y., Xie S., Zhong X. (2013). The positive correlation between *DJ-1* and β-catenin expression shows prognostic value for patients with glioma. Neuropathology.

[15] Ling T., Zhang J., Ding F., Ma L. (2023). Role of growth differentiation factor 15 in cancer cachexia (Review). Oncol. Lett..

[16] Codó P., Weller M., Kaulich K., Schraivogel D., Silginer M., Reifenberger G., Meister G., Roth P. (2016). Control of glioma cell migration and invasiveness by GDF-15. Oncotarget.

[17] Liu B., Zhang B., Qi J., Zhou H., Tan L., Huang J., Huang J., Fang X., Gong L., Luo J. (2023). Targeting *MFGE8* secreted by cancer-associated fibroblasts blocks angiogenesis and metastasis in esophageal squamous cell carcinoma. Proc. Natl. Acad. Sci. USA.

[18] Yamada K., Uchiyama A., Uehara A., Perera B., Ogino S., Yokoyama Y., Takeuchi Y., Udey M.C., Ishikawa O., Motegi S.-I. (2016). MFG-E8 drives melanoma growth by stimulating mesenchymal stromal cell-induced angiogenesis and M2 polarization of tumor-associated macrophages. Cancer Res..

[19] Abd El Atti R.M., Abou Gabal H.H., Osman W.M., Saad A.S. (2013). Insights into the prognostic value of *DJ-1* and MIB-1 in astrocytic tumors. Diagn. Pathol..

[20] Lin J.P., Pan B.C., Li B., Li Y., Tian X.Y., Li Z. (2014). *DJ-1* is activated in medulloblastoma and is associated with cell proliferation and differentiation. World J. Surg. Oncol..

[21] Jin W. (2020). Novel insights into *PARK7* (*DJ-1*), a potential anti-cancer therapeutic target, and implications for cancer progression. J. Clin. Med..

[22] Guo L., Chen Y., Hu S., Gao L., Tang N., Liu R., Qin Y., Ren C., Du S. (2022). *GDF15* expression in glioma is associated with malignant progression, immune microenvironment, and serves as a prognostic factor. CNS Neurosci. Ther..

[23] Park H., Nam K.S., Lee H.J., Kim K.S. (2022). Ionizing radiation-induced *GDF15* promotes angiogenesis in human glioblastoma models by promoting VEGFA expression through p-MAPK1/SP1 signaling. Front. Oncol..

[24] The Human Protein Atlas MFGE8 Expression in Glioma—Pathology Section. https://www.proteinatlas.org/ENSG00000140545-MFGE8/cancer.

[25] Hinkle D.A., Mullett S.J., Gabris B.E., Hamilton R.L. (2011). *DJ-1* expression in glioblastomas shows positive correlation with p53 expression and negative correlation with epidermal growth factor receptor amplification. Neuropathology.

[26] Li Y., Qiu J., Meng Z., Yin S., Ruan M., Zhang W., Wu Z., Ding T., Huang F., Wang W. (2023). MFG-E8 promotes M2 polarization of macrophages and is associated with poor prognosis in patients with gastric cancer. Heliyon.

[27] Fang M., Zhong X.Y., Du B., Lin C.L., Luo F., Tang L.-J., Chen J. (2010). Role of *DJ-1*-induced PTEN down-regulation in migration and invasion of human glioma cells. Chin. J. Cancer.

[28] Malmer B., Feychting M., Lönn S., Ahlbom A., Henriksson R. (2005). p53 genotypes and risk of glioma and meningioma. Cancer Epidemiol. Biomark. Prev..

[29] Kato I., Maita H., Takahashi-Niki K., Saito Y., Noguchi N., Iguchi-Ariga S.M., Ariga H. (2013). Oxidized *DJ-1* inhibits p53 by sequestering p53 from promoters in a DNA-binding affinity-dependent manner. Mol. Cell. Biol..

[30] Jiang Y., Zhou J., Zhao J., Zhang H., Li L., Li H., Chen L., Hu J., Zheng W., Jing Z. (2020). The U2AF2 /circRNA ARF1/miR-342–3p/ISL2 feedback loop regulates angiogenesis in glioma stem cells. J. Exp. Clin. Cancer Res..

[31] Kathagen A., Schulte A., Balcke G., Phillips H.S., Martens T., Matschke J., Günther H.S., Soriano R., Modrusan Z., Sandmann T. (2013). Hypoxia and oxygenation induce a metabolic switch between pentose phosphate pathway and glycolysis in glioma stem-like cells. Acta Neuropathol..

[32] Zhou Q., Li X., Zhou H., Zhao J., Zhao H., Li L., Zhou Y. (2024). Mitochondrial respiratory chain component NDUFA4: A promising therapeutic target for gastrointestinal cancer. Cancer Cell Int..

[33] Guo C., Zhuang Y., Chen Y., Chen S., Peng H., Zhou S. (2020). Significance of tumor protein P53 mutation in cellular process and drug selection in brain lower grade (WHO Grades II and III) glioma. Biomark. Med..

[34] Mei J., Wang T., Xu R., Chen D., Zhang Y. (2021). Clinical and molecular immune characterization of ERBB2 in glioma. Int. Immunopharmacol..

[35] Liu P.C., Lieu A.S., Lin C.J., Tsai H.P., Chai C.Y., Kwan A.L. (2021). High expression of Sp1 is associated with recurrence of meningioma. World Neurosurg..

[36] Kim J.H., Zheng L.T., Lee W.H., Suk K. (2011). Pro-apoptotic role of integrin β3 in glioma cells. J. Neurochem..

[37] Zhang L.Y., Guo Q., Guan G.F., Cheng W., Cheng P., Wu A.H. (2019). Integrin beta 5 is a prognostic biomarker and potential therapeutic target in glioblastoma. Front. Oncol..

[38] Krenciute G., Prinzing B.L., Yi Z., Wu M.F., Liu H., Dotti G., Balyasnikova I.V., Gottschalk S. (2017). Transgenic expression of IL15 improves antiglioma activity of IL13Rα2-CAR T cells but results in antigen loss variants. Cancer Immunol. Res..

[39] Guo K., Song L., Chang J., Cao P., Liu Q. (2020). AEBP1 promotes glioblastoma progression and activates the classical NF-κB pathway. Behav. Neurol..

[40] Tan Z., Zhang Z., Yu K., Yang H., Liang H., Lu T., Ji Y., Chen J., He W., Chen Z. (2022). Integrin subunit alpha V is a potent prognostic biomarker associated with immune infiltration in lower-grade glioma. Front. Neurol..

[41] Scott J., Tsai Y.Y., Chinnaiyan P., Yu H.H.M. (2011). Effectiveness of radiotherapy for elderly patients with glioblastoma. Int. J. Radiat. Oncol. Biol. Phys..

[42] Karsy M., Burnett B., Di Ieva A., Cusimano M.D., Jensen R.L. (2018). Microvascularization of Grade I meningiomas: Effect on tumor volume, blood loss, and patient outcome. J. Neurosurg..

[43] Jovčevska I., Kočevar N., Komel R. (2013). Glioma and glioblastoma—How much do we (not) know?. Mol. Clin. Oncol..

[44] Wang X., Gong Y., Wang D., Xie Q., Zheng M., Zhou Y., Li Q., Yang Z., Tang H., Li Y. (2012). Analysis of gene expression profiling in meningioma: Deregulated signaling pathways associated with meningioma and EGFL6 overexpression in benign meningioma tissue and serum. PLoS ONE.

[45] Wong E., Nahar N., Hau E., Varikatt W., Gebski V., Ng T., Jayamohan J., Sundaresan P. (2019). Cut-point for Ki-67 proliferation index as a prognostic marker for glioblastoma. Asia Pac. J. Clin. Oncol..

[46] Zhao Y., Xu J., Chen B., Cao L., Chen C. (2022). Efficient prediction of Ki-67 proliferation index in meningiomas on MRI: From traditional radiological findings to a machine learning approach. Cancers.

[47] Schmittgen T.D., Livak K.J. (2008). Analyzing real-time PCR data by the comparative CT method. Nat. Protoc..

